# Highly porous Zinc Stannate (Zn_2_SnO_4_) nanofibers scaffold photoelectrodes for efficient methyl ammonium halide perovskite solar cells

**DOI:** 10.1038/srep11424

**Published:** 2015-06-22

**Authors:** Sawanta S. Mali, Chang Su Shim, Chang Kook Hong

**Affiliations:** 1 Polymer Energy Materials Laboratory, Department of Advanced Chemical Engineering, Chonnam National University, Gwangju-S. Korea

## Abstract

Development of ternary metal oxide (TMO) based electron transporting layer (ETL) for perovskite solar cell open a new approaches toward efficient a unique strategy for solid state dye-sensitized solar cells (ssDSSCs). In the present investigation, highly porous zinc tin oxide (Zn_2_SnO_4_) scaffold nanofibers has been synthesized by electrospinning technique and successfully used for methyl ammonium lead halide (CH_3_NH_3_PbI_3_) perovskite sensitized solid state solar cells. The fabricated optimized perovskite solar cell devices exhibited 7.38% power conversion efficiency (PCE) with open circuit voltage (V_OC_) 0.986 V, current density (J_SC_) = 12.68 mAcm^-2^ and fill factor (FF) 0.59 under AM 1.5 G sunlight (100 mWcm^−2^) which is higher than Zn_2_SnO_4_ nanoparticle (η = 2.52%) based perovskite solar cells. This improvement is achieved due to high porosity of Zn_2_SnO_4_ nanofibers and high crystallinity of the nanofibers synthesized at 700 °C. These results are remarkably higher than reported perovskite solar cells based on such type of ternary metal oxide ETLs.

Recently, perovskite solar cell (PSC) based on different organo metal halides and mixed halides have attracted attention as promising efficient solid state solar cells due to their low fabrication cost, easily process ability, easily fine band gap tuning, and variety of mixed halides^1^,^2^,^3^,^4^. Blocking TiO_2_ (Bl-TiO_2_) layer, mesoporous TiO_2_ (mp-TiO_2_), organometallic halide perovskite sensitizers (CH_3_NH_3_PbI_3_ and CH_3_NH_3_PbI_3-x_Cl_x_), hole transporting material (HTM) (spiro-MeOTAD) and counter electrode (Au) are the key components of mesoscopic heterojunction structure perovskite solar cells, while mp-TiO_2_ is absent in planar heterojunction type perovskite solar cells^5^,^6^. This compact Bl-TiO_2_ layer can be deposited by spin or thermal oxidation method. The quality of compact Bl-TiO_2_ play important role in lowering the dark current density and series resistance^7^. Moreover, the incorporation of p-/n-type organic semiconductors in perovskite solar cell is also one of the new configurations of perovskite solar cell^8^,^9^,^10^.

Recently, few reports are available based on Al_2_O_3_^11^, NiO^12^ ZnO^13^ and graphene/TiO_2_^14^ based PSC. We have synthesized atomic layer deposited TiO_2_ passivated 1D TiO_2_ nanorods for CH_3_NH_3_PbI_3_ perovskite nanoparticles sensitization from γ-butyrolactone (GBL) solvent. Such passivated device shows 13.45% power conversion efficiency^15^,^16^. Also, few reports are available based on rutile TiO_2_ nanorods^17^ and anatase nanotubes^18^. On the other hand, there is no substantial reports are available based on ternary metal oxide (TMO) as electron transporting layer (ETL) for PSC. Shin *et al.* reported nano-particulate BaSnO_3_ and Zn_2_SnO_4_ ternary metal oxides (TMO) for dye sensitized solar cells, and demonstrated 6.2%, 6%, power conversion efficiency (PCE) respectively^19^,^20^. Moreover, Zn_2_SnO_4_ nanoparticles have been used for PSC and demonstrated ~7% PCE^21^.

New hierarchical nanostructures, composite of metal oxides or ternary metal oxides will always provide better properties than traditional metal oxides^22^,^23^. The Zn_2_SnO_4_ ternary metal oxide is n-type semiconducting materials having very similar properties with higher band gap 3.7 eV (for anatase TiO_2_ (3.2 eV). However, charge injection and electron diffusion efficiency of this material is much faster than the TiO_2_-based photoanode. On the other hand, the wide band gap (3.7 eV) reduces photobleaching and presents a lower electron-triiodide recombination rate^24^. Moreover, Zn_2_SnO_4_ having high electron mobility of 10–15 cm^2^V^−1^s^−1^ 25.

To the authors best knowledge, there is no single report available based on Zn_2_SnO_4_ nanofibers for perovskite solar cell. In this investigation, we report the synthesis and characterization of Zn_2_SnO_4_ nanofibers by electrospinning method and make use in perovskite solar cells. Further, efforts have been made to increase the scaffold architecture and uniform deposition of perovskite and HTM layer.

## Results

**Figure 1:**
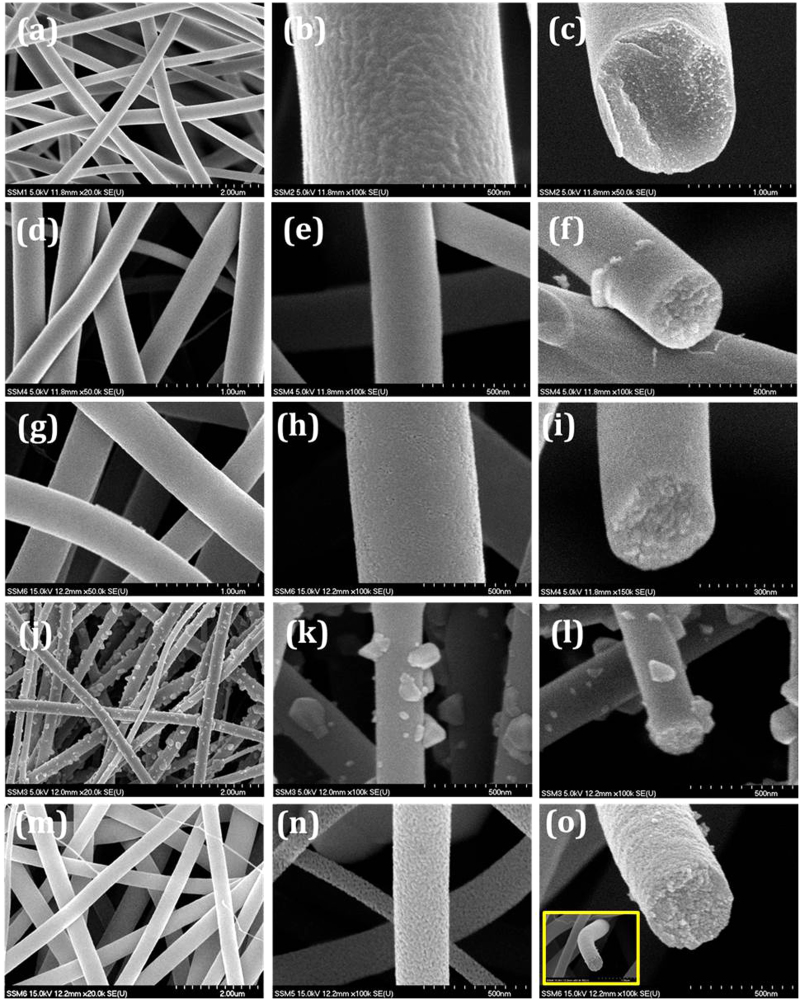
Surface morphology of Zn_2_SnO_4_ nanofibers: (**a-b**) as-spun Zn_2_SnO_4_/PVP composite at different magnifications (**d-e**) annealed at 500 °C, (**g-h**) annealed at 600 °C, (**j-k**) annealed at 670 °C and (**m-n**) annealed at 700 °C. Right hand side images (**c, f, i, l &o**) show respective cross sectional view.

The surface morphology of Zn_2_SnO_4_/PVP nanofibers were characterized by field emission scanning electron microscopy (FESEM). Figure 1 shows FESEM images of as deposited and annealed Zn_2_SnO_4_/PVP composite nanofibers at different temperatures. Figure 1a–c show FESEM images of as deposited Zn_2_SnO_4_/PVP nanofibers at different magnification. From surface morphology of as-deposited Zn_2_SnO_4_/PVP composite nanofibers it is clear that the diameter range of 600–700 nm and several micrometers long in length. Also it is noted that the surface of nanofibers is smooth and compact in nature. The cross sectional image shows perfect circular and solid nanofibers with 700 nm diameter formed at 0.5 ml.h^−1^ feeding rate. Basically, when 17 kV electric field applied between Zn_2_SnO_4_/PVP composite solution and drum, the Zn_2_SnO_4_/PVP fiber stream ejected from a positively charged Talyor cone formed at the nozzle tip, undergoes the solidification followed by phase separation between the organic PVP polymer and inorganic Zn, Sn precursors. In order to remove PVP from precursor and study its architecture, we have annealed as-deposited Zn_2_SnO_4_/PVP composite nanofibers at different temperatures.

The annealing temperature of Zn_2_SnO_4_/PVP was determined by Thermogravimetric analysis (TGA). Figure S1 and Figure S2 show TGA curves of bare PVP and Zn_2_SnO_4_/PVP nanofibers respectively. The weight loss of bare PVP began to occur at approximately 360 °C, and was complete at about 486 °C. Therefore 500 °C annealing temperature is enough for complete decomposition of PVP^26^. The Zn_2_SnO_4_/PVP nanofibers exhibited a three-step process of weight loss, with a total weight loss of 75.3%. The weight loss is a function of temperature: 27% loss from room temperature to 250 °C, followed by 55% between 250 and 325 °C, and finally, a loss of 18.5% that started at about 495 °C and ended at about 750 °C. The first step can be attributed to the loss of *N,N’*-dimethylformamide (DMF) and water evaporation. The second significant weight loss can be attributed to loss by the oxidation of sulfides, and PVP chains are decomposed thermally. Over about 720 °C, there is only a slight weight loss up to 1000 °C, and it is expected that the only material changes that occur at this stage are in the crystal structure. It is clear from the TGA curve that the PVP and organic group were completely removed at 500 °C. Therefore we have annealed our first sample at 500 °C. Figure 1d–f show FESEM images of annealed Zn_2_SnO_4_/PVP nanofibers at 500 °C. After calcination at 500 °C, Zn_2_SnO_4_ nanofibers with relatively rough surface morphology were observed due to thermal decomposition of PVP^27^. Figure 1f shows the cross-sectional images of single Zn_2_SnO_4_ nanofiber calcined at 500 °C. The surface and inner morphology of the fibers is rough.

Figure 1g–i show Zn_2_SnO_4_ nanofibers annealed at 600 °C. It is observed that, the sample annealed at 600 °C shows more compact and rough nature while the diameter of nanofibers have been drastically decreased up to 350 nm. This may be due to complete decomposition of PVP matrix. Figure 1(j–l) show the FESEM micrographs of Zn_2_SnO_4_ nanofibers annealed at 670 °C show porous in nature. Also, surface of nanofibers are swelling and small grains are formed on the surface of nanofibers. These grains are may be due to secondary phases of ZnO. These phases also confirmed by XRD. The cross-sectional image shows around 350 nm diameter with highly porous nature. Figure 1 m-o show the FESEM micrographs of Zn_2_SnO_4_ nanofibers annealed at 700 °C. At a glance it is clear that there is no indication of secondary phases of ZnO. The highly magnified FESEM image shows high porous scaffolds. The cross sectional image also revealed that Zn_2_SnO_4_-700 sample having 350nm in diameter with highly porous scaffold architecture.

**Figure 2:**
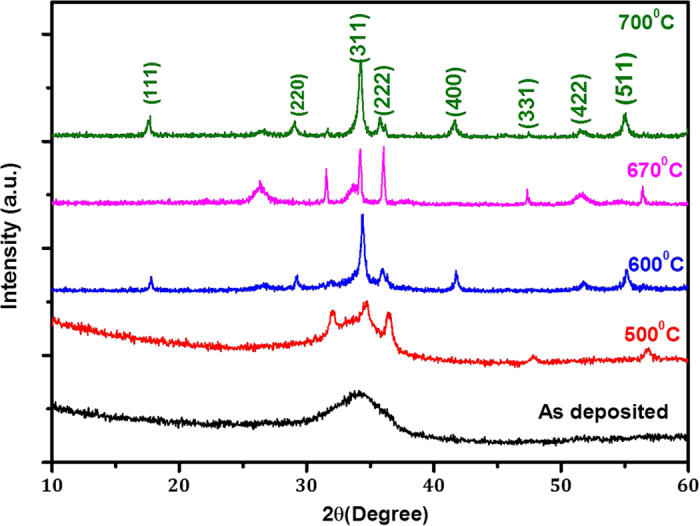
XRD patterns of Zn_2_SnO_4_ nanofibers: annealed at different annealing temperature.

**Figure 3:**
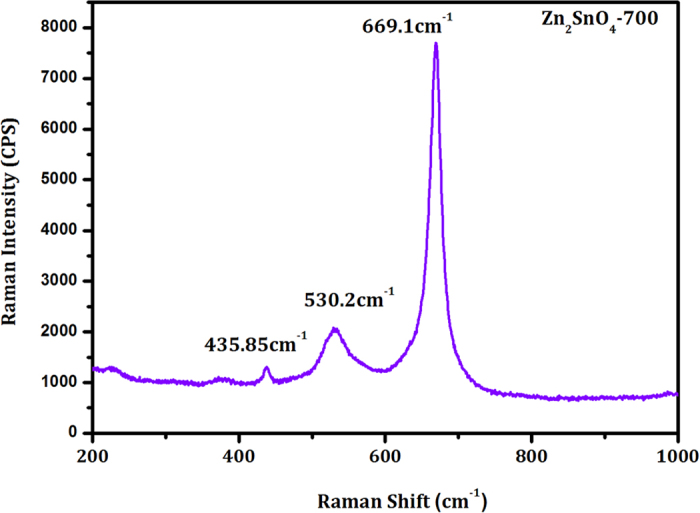
micro-Raman spectrum of Zn_2_SnO_4_-700 nanofibers.

In order to check the crystal structure of these Zn_2_SnO_4_ nanofibers with respect to annealing temperature, we have recorded XRD patterns at different annealed samples. Figure 2 shows the X-ray diffraction (XRD) patterns of Zn_2_SnO_4_ nanofiber after calcination at various temperatures from 500 °C to 700 °C for 1h. Obviously as deposited Zn_2_SnO_4_-PVP nanofiber samples are amorphous in nature. One broad peak was observed at around 30–40°, which originated from the amorphous as-deposited Zn_2_SnO_4_-PVP composite nanofibers. The sample annealed at 500 °C shows that the low crystalline nature of the Zn_2_SnO_4_ nanofibers. However, still there is hump between 30–40°. In order to increase the crystallinity and porosity of the sample, we have further annealed as deposited Zn_2_SnO_4_ nanofibers at 600 °C, 670 °C and 700 °C. Both samples sintered at 600 °C and 670 °C shows crystalline in nature. However, 670 °C sample exhibits mixed phase of ZnO and Zn_2_SnO_4_. On the other hand, the sample annealed at 700 °C exhibits perfect phase of porous Zn_2_SnO_4_ nanofibers. The characteristic peaks of crystalline zinc stannate, that is, the (111), (220), (311), (222), (400), (311), (422) and (511) peaks, of zinc stannate were observed. The estimated lattice parameter was 8.6586 Å, which is in a good agreement with the reported value of 8.6574 Å (JCPDS 24-1470) cubic inverse spinel phase (Supporting Information Table S1)^25^. In order to further confirmation, we have recorded micro-Raman spectrum of Zn_2_SnO_4_-700 sample (Figure 3). The strong Raman shift peaks at 669.1(A1g), 530.2(F2g)and 435.85(Eg) cm^−1^ corresponding to well-known Zn_2_SnO_4_ peaks^25^,^28^,^29^. No additional secondary phase of ZnO is observed. The presence of all three peaks with high intensity indicates the good crystallinity of the Zn_2_SnO_4_-700 nanofibers.

**Figure 4:**
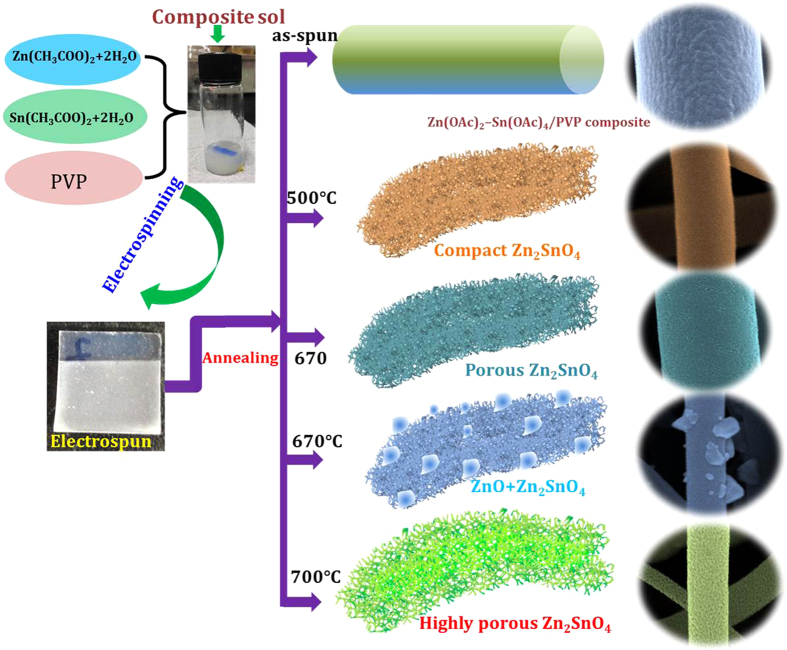
Possible growth mechanism of formation of transformation of compact to porous Zn_2_SnO_4_ nanofibers scaffold.

As per above discussion, we have proposed a possible growth mechanism from as-deposited to compact to highly porous scaffold Zn_2_SnO_4_ nanofibers as shown in Fig. 4. It is well known that, the as deposited nanofibers are highly compact in nature due to homogeneous mixture of Zn, Sn precursor and PVP polymer in DMF solution. When as prepared Zn_2_SnO_4_-PVP nanofibers annealed at 500 °C, the unsaturated carbon backbone and organic composites burned out slowly and pure phase zinc stannate Zn_2_SnO_4_ has been formed. Due to crystallization of Zn and Sn precursors to form crystalline zinc stannate Zn_2_SnO_4_ nanofibers. On the other hand, the sample annealed at 600 °C, Zn, Sn precursors crystalized and PVP polymers with solvents evaporated rapidly results in porous nature of zinc stannate Zn_2_SnO_4_ nanofibers. In both Zn_2_SnO_4_-500 and Zn_2_SnO_4_-600 sample, the polymeric composites decomposes slowly results in crystalline zinc stannate Zn_2_SnO_4_ nanofibers having ~3–4 nm pores. While, in case of the sample annealed at 700 °C, the annealing temperature is quite high and rapidly, therefore the carbon backbone with organic solvents evaporates very drastically compared to rest of the sample. In this process Zn and Sn precursors are crystalized rapidly retaining their highly porous inner morphology. On this fast burned out process, around 15nm pores are formed on the surface of nanofibers. However, the porous nanofibrous framework remains intact even after 700 °C (Fig. 4). In order to confirm the surface chemistry of fabricated Zn_2_SnO_4_ nanofibers, XPS measurements has been performed. As shown in Figure S3, the Zn_2_SnO_4_-700 nanofibers exhibited peaks at 1044.1, 1021.7, 494.71 and 485.55 eV, which could be ascribed to Zn2p_1/2_, Zn2p_3/2_, Sn3d_3/2_ and Sn3d_5/2_, respectively.

**Figure 5:**
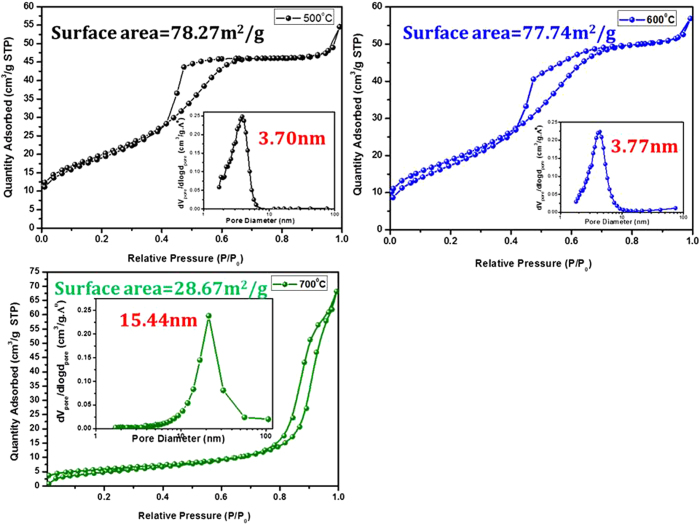
Nitrogen adsorption/desorption isotherms of Zn_2_SnO_4_ nanofibers. Inset plots show respective BHJ pore-size distribution (PSD).

The nanofibers morphology remains intact at higher temperature while, the porosity increased drastically. Moreover, the nanofibers are highly porous scaffold has been formed with ~350 nm diameter. In order to confirm the porosity of these samples, we have recorded BET of Zn_2_SnO_4_-500, Zn_2_SnO_4_-600, and Zn_2_SnO_4_-700 samples. Figure 5 shows the nitrogen adsorption and desorption isotherms and the corresponding pore size distributions plots of Zn_2_SnO_4_ nanofibers annealed at different temperature. The specific surface areas were calculated from the Brunauer-Emmett-Teller (BET) method and the pore size distributions (PSD) were obtained by means of the Barrett-Joyner-Halenda (BJH) equation using the adsorption isotherm branch. The BET specific surface areas of Zn_2_SnO_4_-500 and Zn_2_SnO_4_-600 samples were found to be 78.27 and 77.74 m^2^/g, respectively. However Zn_2_SnO_4_-700 shows drastic decrement in surface area and reduces up to 28.67 m^2^/g. In order to check this anomalous behavior of Zn_2_SnO_4_-700 sample, we have recorded the PSD for all samples. The PSD plots of all samples are represented in respective insets. The Zn_2_SnO_4_-500 and Zn_2_SnO_4_-600 samples exhibited 3.70 nm and 3.77 nm respectively, while Zn_2_SnO_4_-700 sample exhibits 15.44 nm pore size. Such high pores can be attributed to their high porous scaffold interconnected structure compared to rest samples. This high porous scaffold interconnected structure of Zn_2_SnO_4_-700 could provide easy penetration of CH_3_NH_3_PbI_3_/GBL solution and quick crystallization.

**Figure 6:**
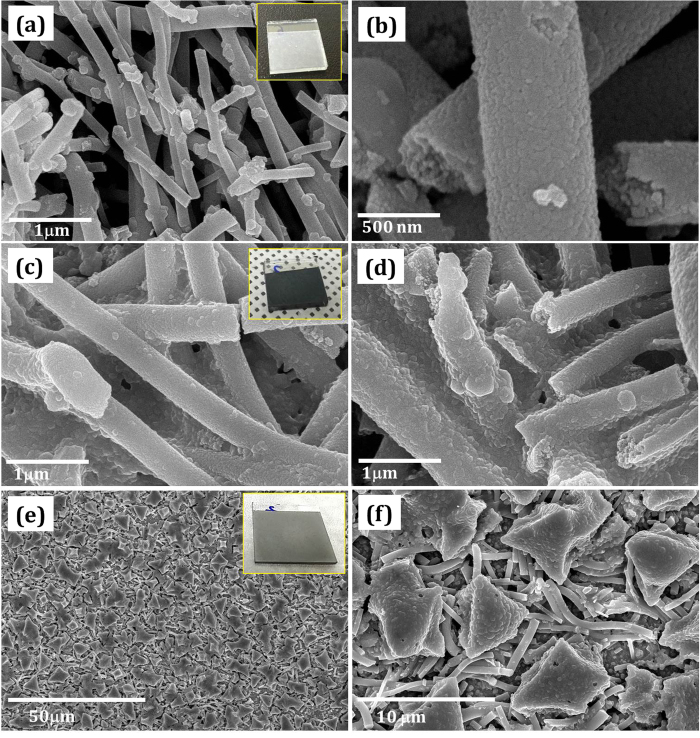
Surface morphology of Zn_2_SnO_4_/perovskite solar cell at different stages (**a-b**) FESEM images of Zn_2_SnO_4_-700 nanofibers spin coated and annealed on FTO/Bl-ZSO substrate (**c-d**) after deposition of CH_3_NH_3_PbI_3_ on Zn_2_SnO_4_ nanofibers. (**e-f**) after deposition of spiro-MeOTAD HTM material. Photographs at each step are shown in inset.

In this investigation, we focused on Zn_2_SnO_4_-700 due to scaffold nano-architecture, while Zn_2_SnO_4_-500, Zn_2_SnO_4_-600 and Zn_2_SnO_4_-670 samples have also been investigated for comparison. The paste of Zn_2_SnO_4_ nanofibers has been prepared using terpeniol and ethyle cellulose in ethanol solvent and spin coated on to Bl-ZSO/FTO sample. The Zn_2_SnO_4_-700 paste was prepared with the help of paste mixer (DAE WHA TECH, PDM-300) and spin coated on FTO/Bl-ZSO substrate at desired speed. The thickness and uniformity of Zn_2_SnO_4_ electrodes were optimized using solution viscosity and spin coating speed. (Please check supporting information Figure S4). The deposited samples were further sequentially annealed at 500 °C for 30 min in order to complete evaporation of organic solvents. Figure 6 shows surface morphology of Zn_2_SnO_4_-700 nanofibers before and after perovskite deposition. After gradual annealing process, white colored adherent Zn_2_SnO_4_ nanofiber film has been form (Inset of Fig. 6(a)). From Figure 6(a–b), it is clear that, the Zn_2_SnO_4_-700 scaffold nanofibers are deposited onto FTO/Bl-ZSO substrate. However, it is observed that, these Zn_2_SnO_4_-700 nanofibers are fragmented instead of long nanofibers. After calcination process, these nanofibers were used for CH_3_NH_3_PbI_3_/GBL solution casting. The CH_3_NH_3_PbI_3_/GBL solution was spin coated and dried on hot plate in order to evaporate GBL solvent. After evaporation of GBL solvent, dark brown colored CH_3_NH_3_PbI_3_ film has been formed (Inset Fig. 6 (a)). Figure 6c–d show the FESEM micrographs of CH_3_NH_3_PbI_3_+Zn_2_SnO_4_ composite nanofibers. The CH_3_NH_3_PbI_3_ nanoparticles are agglomerated onto surface of Zn_2_SnO_4_ nanofibers. The spiro-MeOTAD HTM material has been spin coated at 3000 rpm. Figure 6 (e–f) show typical SEM micrographs of CH_3_NH_3_PbI_3_+Zn_2_SnO_4_ composite nanofibers after spiro-MeOTAD deposition. The large size islands of spiro-MeOTAD HTM material have been covered onto nanofibrous architecture. These samples were further used for gold contact by thermal evaporation.

**Figure 7:**
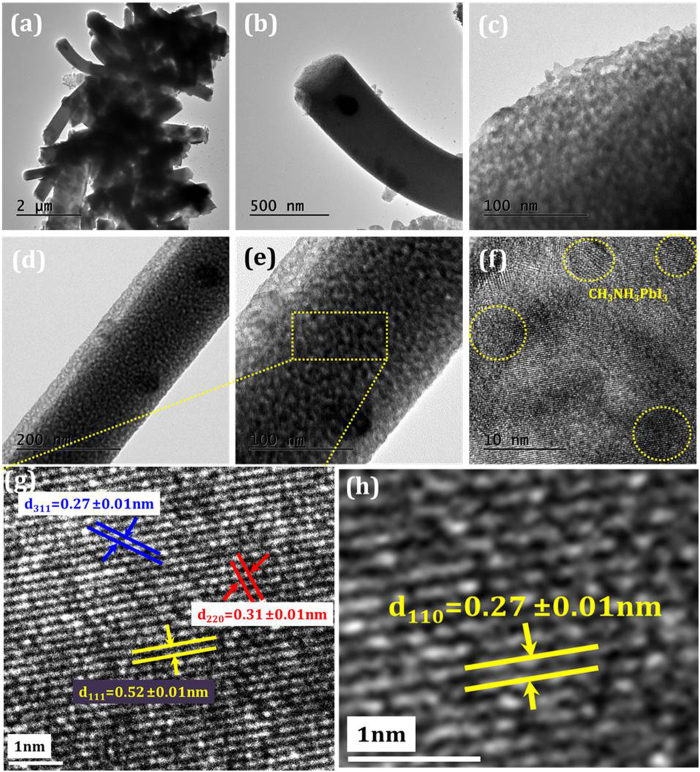
Transmission electron microscopy: (**a**,**b**) TEM micrographs of CH_3_NH_3_PbI_3_+Zn_2_SnO_4_ sample at different magnification (**c**) highly magnified TEM image of CH_3_NH_3_PbI_3_+Zn_2_SnO_4_, (**d**) TEM image of single CH_3_NH_3_PbI_3_+Zn_2_SnO_4_ nanofiber (**e**) Highly magnified TEM image of CH_3_NH_3_PbI_3_+Zn_2_SnO_4_ (**f**) HRTEM image, (**g**) and (**h**) lattice fringes of selected area of Zn_2_SnO_4_ and CH_3_NH_3_PbI_3_ respectively.

**Figure 8:**
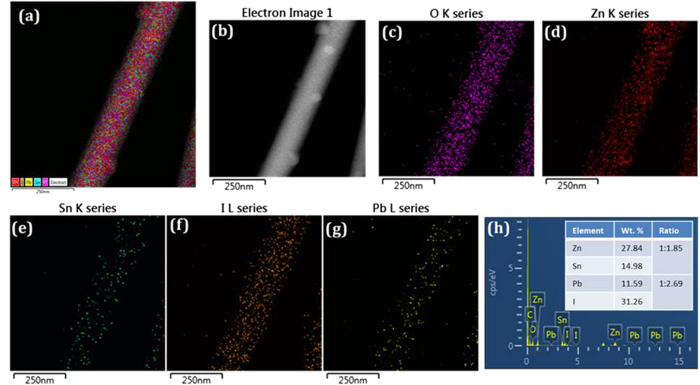
Compositional analysis of CH_3_NH_3_PbI_3_ decorated Zn_2_SnO_4_ nanofiber scaffold: (**a**) Plane view of STEM micrographs of CH_3_NH_3_PbI_3_ decorated Zn_2_SnO_4_ nanofibers and EDS mapping with respective colors (**b**) STEM micrograph (**c**)oxygen (**d**) zinc, (**e**) tin (**f**) iodine, (**g**) lead and (**h**) EDS spectrum of the CH_3_NH_3_PbI_3_ decorated Zn_2_SnO_4_ nanofibers sample. Inset table shows obtained Zn:Sn and Pb:I atomic ratio.

The prepared Zn_2_SnO_4_ nanofibers/Bl-ZSO/FTO substrates were used for CH_3_NH_3_PbI_3_ deposition. Figure 7 show the TEM micrographs of CH_3_NH_3_PbI_3_ loaded Zn_2_SnO_4_-700 nanofiber sample. The diameter of Zn_2_SnO_4_ nanofibers is around 350 nm (Fig. 7(a)), which is well agreement with FESEM data. The highly porous Zn_2_SnO_4_ nanofibers were clearly observed in the TEM analysis. The highly magnified TEM image shows (Fig. 7e), the Zn_2_SnO_4_ nanofibers were composed of nanocrystallites with sizes ranging from 15 to 20 nm. The growth directions for the nanofibers were determined from high resolution TEM (HRTEM) as shown in Fig. 7(f). Lattice images are clearly observed in Fig. 7(g), indicating that Zn_2_SnO_4_ single grains are highly crystalline. The interplanar lattice spacing along the (220), (311) and (111) planes indicated by red, blue and yellow lines are found 0.31 ± 0.01 nm, 0.27 ± 0.01 nm, and 0.52 ± 0.01 nm respectively, which is consistent with the cubic inverse spinel phase crystal structure of Zn_2_SnO_4_ (Fig. 7(g)). The synthesized CH_3_NH_3_PbI_3_ nanoparticles are crystalline in nature with ~7nm in diameter as shown in Fig. 7g. The calculated lattice spacing along d_110_ = 0.27 ± 0.01 nm confirming the tetragonal phase (Fig. 7(h)). However, even though we have deposited CH_3_NH_3_PbI_3_ onto these porous nanofibers, but it shows low crystallinity. Such type of behavior has been observed due to high porous scaffold nature of Zn_2_SnO_4_-700 nanofibers. In order to confirm the CH_3_NH_3_PbI_3_ we have analyzed these samples by EDS elemental mapping. Figure 8 shows the STEM and EDS mapping of CH_3_NH_3_PbI_3_ loaded Zn_2_SnO_4_ nanofibers. The EDS mapping of each elements confirmed that the Zn_2_SnO_4_ + CH_3_NH_3_PbI_3_ composites have all O (magenta color dots), Zn (red color dots), Sn (green color dots), I (orange color dots) and Pb (yellow color dots) elements. The composition of the elements shows excellent stoichiometry throughout the surface (Fig. 8a–g). Figure 8h shows EDS spectrum of CH_3_NH_3_PbI_3_/Zn_2_SnO_4_ sample. It is also noted that the atomic wt. % ratio of Pb and I is 1:3 and composition of Zn_2_SnO_4_ fibers revealed that the Zn/Sn chemical composition ratio was approximately 2:1 confirms the stoichiometry of the deposited CH_3_NH_3_PbI_3_ as well as Zn_2_SnO_4_ material. The crystal structure of synthesized CH_3_NH_3_PbI_3_ perovskite has also been confirmed by XRD analysis (Figure S5).

**Figure 9:**
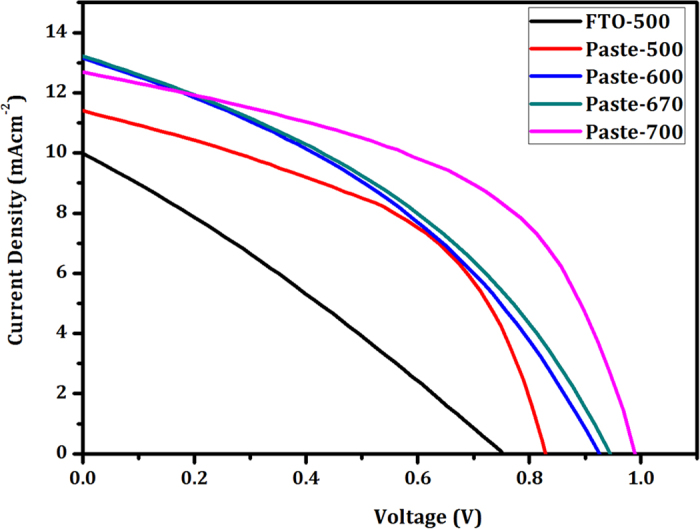
J-V curves of CH_3_NH_3_PbI_3_ sensitized Zn_2_SnO_4_ nanofibers scaffold based perovskite solar cells measured under 100 mWcm^−2^ illumination.

The CH_3_NH_3_PbI_3_/Zn_2_SnO_4_ loaded samples were used for solar cell application. Figure 9 shows J-V curves of perovskite solar cells based on different Zn_2_SnO_4_ photoelectrodes. The direct deposited and annealed at 500 °C Zn_2_SnO_4_ nanofiber based perovskite device shows short-circuit current density (J_SC_) 9.95 mAcm^−2^, open-circuit voltage (V_OC_) 0.752 V, fill factor (FF) 0.29 leading to power conversion efficiency (PCE) η = 2.16% (Fig. 9). This sample shows very low FF due to direct contact between FTO and HTM material (Figure S4). Moreover, the HTM material has been agglomerated onto Zn_2_SnO_4_ nanofibers. In order to improve the compactness of photoelectrode, it is necessary to make compact film by two approaches. One is to anneal at higher temperature or second is to prepare paste. In the present case higher temperature >600°C annealing is not possible for FTO substrate. Therefore, we have decided to prepare Zn_2_SnO_4_ paste and deposit by spin coating. The paste prepared from sample Zn_2_SnO_4_-500 sample exhibits V_OC _= 0.829 V, J_SC _= 11.40 mAcm^−2^, FF = 0.49 leading to η = 4.63%. This drastic increment in fill factor is only due to compactness of Zn_2_SnO_4_ sample and uniform coating of spiro-MeOTAD. Also these Zn_2_SnO_4_ nanofibers are amorphous in nature. The Zn_2_SnO_4_-600 sample shows drastic increment in V_OC_ up to 0.928 V. This increment is may be due to avoiding spiro-MeOTAD and FTO contact. If spiro-MeOTAD is directly in contact with FTO substrate, then cell exhibits ohmic behaviour. Moreover, little increment has also been observed in the J_SC_ = 13.11 mAcm^−2^, FF = 0.39 and η = 4.74%. The Zn_2_SnO_4_-670 photoelectrode exhibits η = 4.84% with V_OC_ = 0.941 V, J_SC_ = 13.19 mAcm^-2^ and FF = 0.39. However, in this case also the FF is not good. Therefore, the thickness and uniform deposition of Zn_2_SnO_4_ has been optimized by spin coating speed. Figure S4 show FESEM micrographs of Zn_2_SnO_4_-700 nanofibers deposited at different spin coating conditions with respective to their cross sectional micrographs. Here, we have varied the spin coating speed from 2000 rpm to 5000 rpm and used for perovskite deposition followed by spiro-MeOTAD HTM coating. Please note that, we have deposited HTM layer at 3000 rpm (30 sec) and 80 nm gold contacts deposited by thermal evaporation. The spin coating speed has been varied from 2000 rpm to 5000 rpm with 500 rpm interval. It is observed that, as spin coating rate increases, the uniformity of Zn_2_SnO_4_-nanofibers as well as HTM coating becomes uniform. On the other hand the thickness of the photoelectrodes has also been decreases. This is good indication for well optimization of thickness of photoelectrodes. Figure S4 (a–h) shows the respective photographs of deposited Zn_2_SnO_4_ photoelectrodes. The obtained solar cell parameters are summarized in Table S2. At a glance, it is observed that, the sample deposited at lower spin coating speed exhibits low V_OC_, low FF. This is might be due to higher film thickness and random coating of HTM layer. This may causes ohmic behaviour of the perovskite solar cells due to direct contact between HTM and FTO substrate. The Zn_2_SnO_4_-700 (Zn_2_SnO_4_-700 paste deposited at 5000 rpm) sample exhibits V_OC_ = 0.986V, J_SC_ = 12.68 mAcm^−2^, FF = 0.59 leading to η = 7.39%. This improvement in V_OC_ with respect to spin coating thickness revealed that decline in the backflow of electrons from CB of Zn_2_SnO_4_ to CH_3_NH_3_PbI_3_ and HTM. For comparison, we also have synthesized Zn_2_SnO_4_ nanoparticles by hydrothermal method. Figure S6 show typical SEM micrographs, XRD pattern and J-V performance of Zn_2_SnO_4_ nanoparticles based perovskite solar cell. Approximately, ~30 nm particle size of Zn_2_SnO_4_ nanoparticles based device shows V_OC_ = 0.731 V, J_SC_ = 8.86 mAcm^-2^, FF = 0.39with PCE = 2.52%. However, in this case still optimization of photoelectrodes thickness is needed.

**Figure 10:**
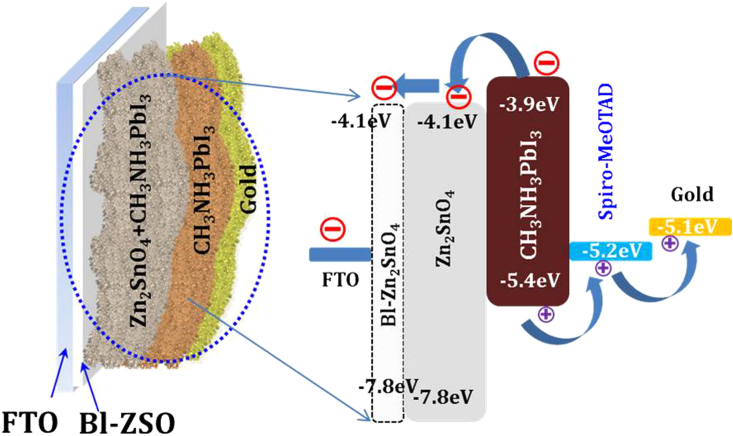
Schematic representation of energy level diagram of Zn_2_SnO_4_ nanofibers based perovskite solar cells. The energy level values are taken as per previous literature.

Based on the above discussion, we have proposed possible solar cell mechanism of the fabricated Zn_2_SnO_4_ nanofiber based perovskite solar cells. The working principle of the perovskite solar cell is as shown in Figure 10. The CH_3_NH_3_PbI_3_/ Zn_2_SnO_4_ onto FTO coated substrate acts as a working electrode. Here, CH_3_NH_3_PbI_3_ nanoparticles acts as the absorber layer which sandwiched between an electron transport layer (ETL) i.e. Zn_2_SnO_4_ and hole transport layer (HTL) i.e. spiro-MeOTAD and gold contact act as counter electrode. When this device illuminates to photon energy following process takes place step by step. Here the light absorbing layer CH_3_NH_3_PbI_3_ absorbs the photon energy in the visible region to create an electron–hole pair.
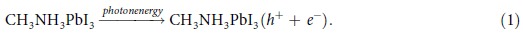


Due to band alignment of Zn_2_SnO_4_ and CH_3_NH_3_PbI_3_, the generated electrons will be transferred immediately to the conduction band (CB) of Zn_2_SnO_4_, while holes will transfer through spiro-MeOTAD via hopping mechanism. Since, CB of Zn_2_SnO_4_ is higher than CH_3_NH_3_PbI_3_.
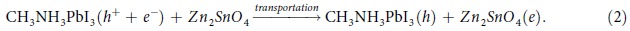


The transferred electrons subsequently flow from FTO to external circuit to produce electricity.

**Figure 11:**
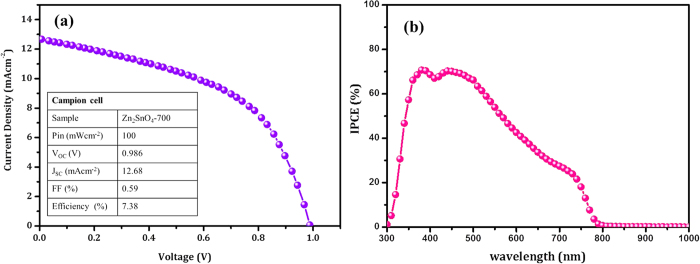
Photovoltaic properties of champion cell. (**a**) J-V curve of Zn_2_SnO_4_-700 based champion cell. The Zn_2_SnO_4_-700 photoelectrode deposited at 5000 rpm (45 sec), HTM layer 3000 (30 sec). (**b**) IPCE spectrum of Zn_2_SnO_4_-700 based perovskite solar cells. The IPCE data was collected under the constant energy DC mode with delay time 10 ms under 50 μWcm^−2^ light intensity.

Figure 11(a) shows the J-V curve of champion cell recorded for Zn_2_SnO_4_-700 sample. The solar cell parameters were summarized in Table-1. The highest PCE of 7.38% has been achieved due to high V_OC_ = 0.986V and higher current density 12.68 mAcm^−2^. It is also observed that, the FF = 0.59, which is higher than the rest Zn_2_SnO_4_ samples. This enhancement is mainly due to the well covering of Zn_2_SnO_4_-700 sample thought out the surface which hinders the direct contact between FTO and HTM. In the present case the improvement in PCE is mainly ascribed to higher V_OC_ and FF parameters. In order to confirm this, we have also recorded IPCE data of the same sample (Fig. 11(b)). The photocurrent generation starts at ~750 nm, in agreement with the band gap of the CH_3_NH_3_PbI_3_ and reached up to ~70% IPCE in the visible spectrum. The plot shows ~70% IPCE in the 400–500 nm wavelength region; however, the IPCE response drastically decreases after 500 nm to 750 nm wavelength. This indicates that, there might be possibility for high recombination rate due to randomly dispersed Zn_2_SnO_4_ nanofibers.

**Figure 12:**
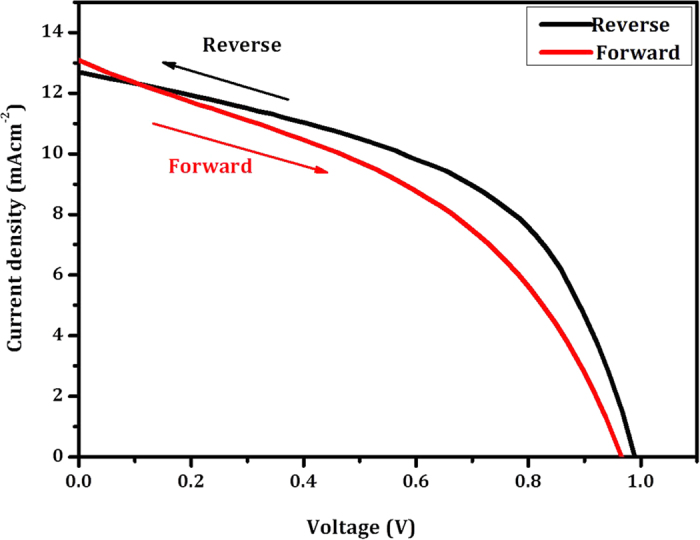
Hysteresis analysis of Zn_2_SnO_4_-700 sample. Photovoltaic performance was measured by forward and reverse scans with 10 mV voltage steps and 50 ms delay times under AM 1.5 G illumination.

The hysteresis behaviour of fabricated Zn_2_SnO_4_-700 nanofiber based perovskite device has been carried out in forward and reverse scan mode. The scanning delay was kept 40 ms for this measurement. Figure 12 shows typical J-V measurements of Zn_2_SnO_4_-700 based perovskite solar cells. The forward scan exhibits V_OC_ = 0.963V, J_SC_ = 13.09 mAcm^-2^, FF = 0.41 with η = 5.17%. However, the reverse scan exhibits η = 7.38% with V_OC_ = 0.986 V, J_SC_ = 12.68 mAcm^-2^ and FF = 0.59. These hysteresis results are summarized in Table-2. In the present case our sample shows hysteresis behaviour mainly due to thickness of the Zn_2_SnO_4_ photoelectrode and random deposition of nanofibers with perovskite. This problem can be eliminated by optimizing the diameter of the Zn_2_SnO_4_ nanofibers and thickness of photoelectrode. Moreover, our sample shows lower fill factor <60%, due to weakly bounded CH_3_NH_3_PbI_3_ nanoparticles on to Zn_2_SnO_4_ scaffold which suffers from weaker absorption. Therefore we suggest that, more compact layer of CH_3_NH_3_PbI_3_ can be enhancing current density and fill factor of the perovskite solar cell. This study is presently underway in our laboratory.

## Conclusions

In summary, we have successfully demonstrated the first use of the ternary Zn_2_SnO_4_ nanofibers for the CH_3_NH_3_PbI_3_ based solid state perovskite solar cell. The synthesized nanofibers with different surface area (Zn_2_SnO_4_ -500, 78.27 m^2^/g, Zn_2_SnO_4_ -600 77.74 m^2^/g and Zn_2_SnO_4_ -700 28.67 m^2^/g) and different pore size (Zn_2_SnO_4_-500 = 3.7 nm, Zn_2_SnO_4_-600 = 3.77 nm and Zn_2_SnO_4_-700 = 15.44 nm) have been controlled by annealing process. Further, the uniform deposition of Zn_2_SnO_4_-700 nanofibers has been optimized and used for CH_3_NH_3_PbI_3_ based solid state perovskite solar cell. Our results revealed that, Zn_2_SnO_4_-700 scaffold like sample exhibits the Voc and fill factor increased with the increasing spin coating rate. In other word, with decrease in thickness of the Zn_2_SnO_4_-700 scaffold layer. Our champion cell demonstrates the best performance of 7.38% PCE with FF = 0.59, J_SC_ = 12.68 mAcm^−2^. However, we expect more PCE for more accurate optimization of diameter and thickness of Zn_2_SnO_4_ photoelectrodes (Tables 1 and 2).

## Methods

### Preparation of blocking layer ZnSnO4 (Bl-ZSO)

Initially, laser pattern F-doped SnO_2_ (FTO) substrates (ML20-PL-R, Kortherm Science), were cleaned by sonication in soap, ethanol and isopropanol followed by the plasma treatment before coating the compact layer. The ZSO-based perovskite solar cells, a Zn_2_SnO_4_ blocking layer (Bl-ZSO) was deposited on the patterned FTO substrate at room temperature by spin coating a solution containing ZnCl_2_ and SnCl_2_ (Zn/Sn ratio = 2) at 3000 rpm for 30 sec, followed by annealing at 350 °C for 10 min under an ambient atmosphere. Then, Bl-Zn_2_SnO_4_/FTO samples were used for electrospinning, followed by annealing at 500 °C for 30 min under an ambient atmosphere.

### Preparation of Zn_2_SnO_4_ nanofibers

Poly(vinyl pyrrolidone) (PVP, Mw = 1,300,000 mol/g), Zn(CH_3_COO)_2_·2H_2_O (99%+) and Sn(CH_3_COO)_4_ (99%+) were purchased from Aldrich. Anhydrous N,N-dimethylformamide (DMF) was obtained from J.T Baker. The chemical reagents were used without further purification. In typical experiment, the Zn_2_SnO_4_ nanofibers were synthesized by electrospinning technique. The precursor solution for electrospinning process was prepared by dissolving 1.756 g of Zn(CH_3_COO)_2_·2H_2_O, 1.42 g of Sn(CH_3_COO)_4_ and 1.3 g of PVP in 7.2 ml of DMF solvent. The homogeneous sol was prepared by overnight stirring at room temperature. The above prepared electrospinning solution was carefully sucked into a 5 ml glass syringe and fixed horizontally arranged electrospinning equipment (SGE analytical Science). The positive electrode was connected to the needle of the syringe containing precursor solution. The drum rotating speed (400 rpm) and the distance between the needle tip and grounded collector (15 cm) was kept constant. The feeding rate was 0.5 mlh^−1^ controlled by a syringe pump (KDS-100, KD Scientific). At this point, an electric field potential of 17 kV was applied between the needle tip and a grounded collector at a distance of 15 cm. The electron spun Zn_2_SnO_4_/PVP composite nanofibers have been deposited on FTO substrate as well as Al foil. The prepared nanofibers were further calcined at 500 °C, 600 °C, 670 °C and 700 °C for each 1hr in air to remove the organic constituents of the PVP polymer matrix and recorded respective XRD. Please note that, FTO substrates are not stable after 500 °C, therefore we have only used prepared nanofibers on Al foil and used for 600 °C, 670 °C and 700 °C annealing temperatures. The thermal annealing temperature was optimized by TGA analysis (Supporting Information Figure S1, Figure S2). The crystallinity and composition of the nanofibers were confirmed by XRD, TEM and EDS mapping analysis.

The annealed nanofibers were further used for Zn_2_SnO_4_ paste. The Zn_2_SnO_4_ paste was prepared using ethyl cellulose and terpineol solution in ethanol solvent. The prepared Zn_2_SnO_4_ paste was spin coated on Bl-ZSO/FTO substrate. The organic solvents were evaporated by annealing process at 500 °C for 30 min. However, the thicknesses, transparency and uniformity of Zn_2_SnO_4_ photoelectrodes were optimized by spin coating speed and the viscosity of Zn_2_SnO_4_ paste by addition of ethanol solvent (Supporting Information Figure S4).

### Preparation of Zn_2_SnO_4_ nanoparticles

The Zn_2_SnO_4_ nanoparticles were fabricated by hydrothermal method. In a typical experiment Zn(NO_3_)_2_·6H_2_O (Aldrich) and SnCl_4_·5H_2_O (Aldrich) were dissolved in an equal volume of water and ethanol. The white coloured viscous sol was obtained by drop wise addition of 1 M NaOH. The viscous sol was transferred to a teflon-lined autoclave, and then kept in an oven at 180 °C for 12 h. The dried and washed precipitate was dispersed in terpineol, and spin-coated on the Bl-ZSO, followed by gradually annealing at 500 °C for 30 min.

The prepared Zn_2_SnO_4_ photoelectrodes were further treated with ZnO treatment. For the ZnO treatment, the ZSO photoelectrodes were immersed in a zinc acetate solution (0.05 m) in an ethanol bath for 30 min at 50 °C. After the films were rinsed with ethanol and air dried and sintered at 500 °C for 1 h.

## Additional Information

**How to cite this article**: Mali, S. S. *et al.* Highly porous Zinc Stannate (Zn_2_SnO_4_) nanofibers scaffold photoelectrodes for efficient methyl ammonium halide perovskite solar cells. *Sci. Rep.* **5**, 11424; doi: 10.1038/srep11424 (2015).

## Supplementary Material

Supplementary Information

## Figures and Tables

**Table 1:** Solar cell parameters of the solid-state perovskite solar cells based on Zn _2_SnO_4_ photoelectrodes deposited at different condition.

Sample	V_**OC**_ (V)	J_SC_ **(mAcm**^−2^)	FF (%)	η (%)
As-spun-500$	0.752 ± 0.02	9.95 ± 0.43	0.29	2.16 ± 0.12
Zn_2_SnO_4_-500	0.829 ± 0.01	11.40 ± 0.35	0.49	4.63 ± 0.23
Zn_2_SnO_4_-600	0.928 ± 0.01	13.11 ± 0.36	0.39	4.74 ± 0.45
Zn_2_SnO_4_-670	0.941 ± 0.01	13.19 ± ±0.31	0.39	4.84 ± 0.45
Zn_2_SnO_4_-700*	0.946 ± 0.01	13.21 ± 0.22	0.41	5.12 ± 0.35
Zn_2_SnO_4_-700 Champion cell	0.986 ± 0.01	12.68 ± 0.33	0.59	7.38 ± 0.36
Zn_2_SnO_4_ nanoparticles	0.731 ± 0.01	08.86 ± 0.33	0.39	2.52 ± 0.30

All measurements were measured at room temperature.

^$^Direct deposited on FTO substrates.

^*^Thickness has been optimized in terms of spin coating rate.

**Table 2:** Hysteresis solar cell studies of Zn2SnO4-700 photoelectrode.

Sample	Scan mode	V_OC_ (V)	J_SC_ (mAcm^−2^)	FF	η (%)
Zn_2_SnO_4_-700*	Forward	0.963	13.09	0.41	5.17
	Reverse	0.986	12.68	0.59	7.38

Table 2. Hysteresis solar cell studies of Zn2SnO4-700 photoelectrode. Device configuration: FTO/Bl-ZSO/Zn2SnO4-700+CH3NH3PbI3/spiro-MeOTAD/Au. The J-V characteristics measured under AM 1.5G. condition with the input solar power Pin of 100 mWcm−2 (40 ms scanning delay). The J-V characteristics measured under AM 1.5G condition with the input solar power Pin of 100 mWcm−2 (40 ms scanning delay).
