# Supplementary material for: Highly porous Zinc Stannate (Zn2SnO4) nanofibers scaffold photoelectrodes for efficient methyl ammonium halide perovskite solar cells
Source: Sci Rep. 2015 Jun 22;5:11424. doi: 10.1038/srep11424 (PMC4476148; doi:10.1038/srep11424)
Supplement: Supplementary Information [file srep11424-s1.pdf]

Supporting Information

Highly porous Zinc Stannate (Zn_2SnO_4) nanofibers scaffold photoelectrodes for efficient methyl ammonium halide perovskite solar cells

Sawanta S. Mali, Chang Su Shim and Chang Kook Hong*

*Polymer Energy Materials Laboratory, Department of Advanced Chemical Engineering, Chonnam National University, Gwangju-S. Korea

Experimental Details:

Preparation of methylammonium lead iodide ($CH_3NH_3PbI_3$)

Methylammonium lead iodide ($CH_3NH_3PbI_3$)was synthesized as per our previous report [1]. In typical experiment, drop wise addition of hydroiodic acid (aqueous, 57 wt %, Sigma-Aldrich) to a solution of methylamine (aqueous, 40 wt %, TCI Chemicals) in an ice bath. The ice-cold solution was stirred for 2 hr, and the solvent was evaporated using a rotary evaporator (95mbar vacuum, 400rpm rotation). The white coloured product was dissolved in ethanol and recrystallize using diethyl ether. Fresh white coloured crystals were washed three times using diethyl ether and dried in vacuum for 24h. The resulting white solid product was further used for $CH_3NH_3PbI_3$ synthesis.

The $CH_3NH_3PbI_3$precursor solution was prepared by dissolving equimolar amounts of CH_3NH_3I and lead iodide (PbI_2) (Aldrich, 99.999%) in anhydrous γ-butyrolactone (GBL) (40% by weight, Sigma-Aldrich) at 60°C and stirred 12h. The prepared yellow colored solution was filtered by twice using syringe filter (Whatman GD/X PVDF pore size 0.45μm). The clear yellow solution was dripped on top of the ZSO nanofibrous photoelectrode, and the film was soaked for 1 min and then spun at 2500 rpm for 45s and 3500rpm for 45s with one ramp rate. The GBL solvent was evaporated on a hot plate at 95°C for 10 min to form crystalline$CH_3NH_3PbI_3$. The hole transport material (HTM) was prepared by as per our previous report [1]. The prepared spiro-MeOTADHTM solution was spin-coated on the FTO/Bl-ZSO/Zn_2SnO_4-NF+$MAPbI_3$ substrate at 3,000 r.p.m for30s. Then the substrates were transferred to a vacuum chamber and evacuated to a pressure of 2×10^{-6} mbar. For the counter electrode, a 80 nm thick Au contacts were deposited on the top of the HTM over

layer by a thermalevaporation (growth rate ~0.5Å/s). Theactive area of this electrode was fixed at 0.09cm^2. The active area was calculated as per gold and laser pattern cross-sectional area.

Reference:

[1] Mali, S. S., Shim, C. S., Park, H. K., Heo J., Patil, P. S. & Hong, C. K. Ultrathin Atomic Layer Deposited TiO_2 for Surface Passivation of Hydrothermally Grown 1D TiO_2 Nanorod Arrays for Efficient Solid-State Perovskite Solar Cells, *Chem. Mater.*, 27, 1541–1551 (2015)

Characterizations:

The top-surface and cross-sectional images were recorded by a field emission scanning electron microscope (FESEM; S-4700, Hitachi). Transmission electron microscopy (TEM) micrographs, selected area electron diffraction (SAED) patter and high-resolution transmission electron microscopy (HRTEM) images were obtained by JEOL JEM-2100F (Field Emission Electron Microscope) operated at 200KV. The TEM sample was prepared by drop casting of ethanolic dispersion of sample onto a carbon coated Cu grid. X-ray diffraction (XRD) measurements were carried out using a D/MAX Uitima IIIXRD spectrometer (Rigaku, Japan) with CuKα line of 1.5410 Å. The elemental information regarding the deposited samples were analyzed using an X-ray photoelectron spectrometer (XPS) (VG Multilab 2000-Thermo Scientific, USA, K-Alpha) with a multi-channel detector, which can endure high photonic energies from 0.1 to 3 keV.

The cells were illuminated using a solar simulator at AM 1.5 G for 10 s, where the light intensity was adjusted with an NREL-calibrated Si solar cell with a KG-5 filter to 1 sun intensity (100 mW cm^{-2}). The IPCE spectra were measured as a function of wavelength from 300 to 1000 nm on the basis of a Spectral Products DK240 monochromator.

Figure S1TGA curve of bare PVP nanofibers.

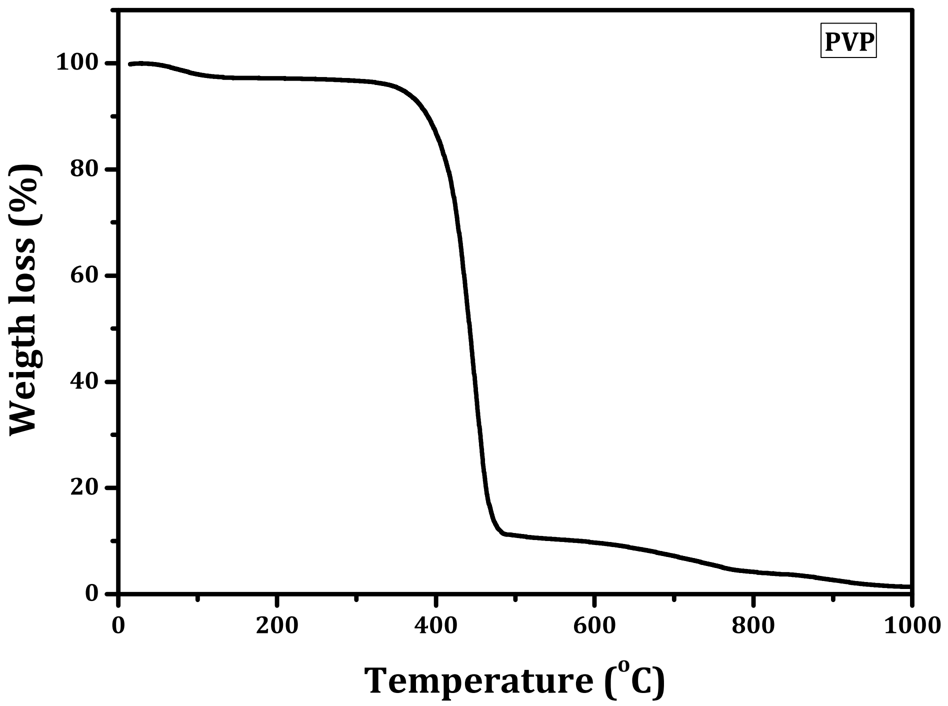

Figure S2TGA curve of Zn_2SnO_4/PVP composite nanofibers.

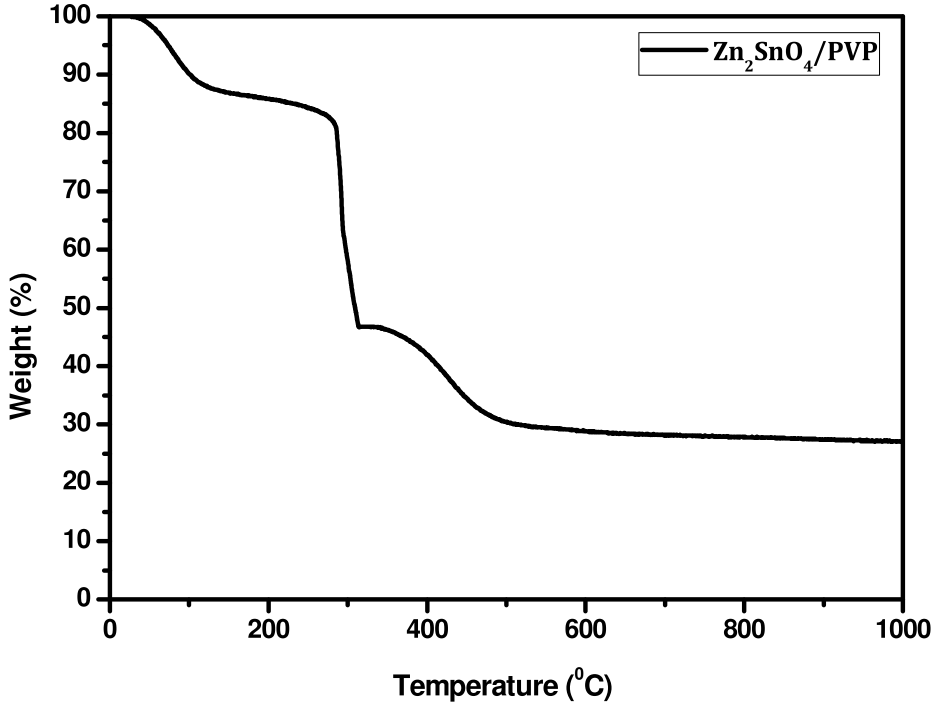

Figure S3 XPS spectra of Zn_2SnO_4-700 nanofibers. (a) Zn2p core level spectrum (b) Sn2d core level spectrum.

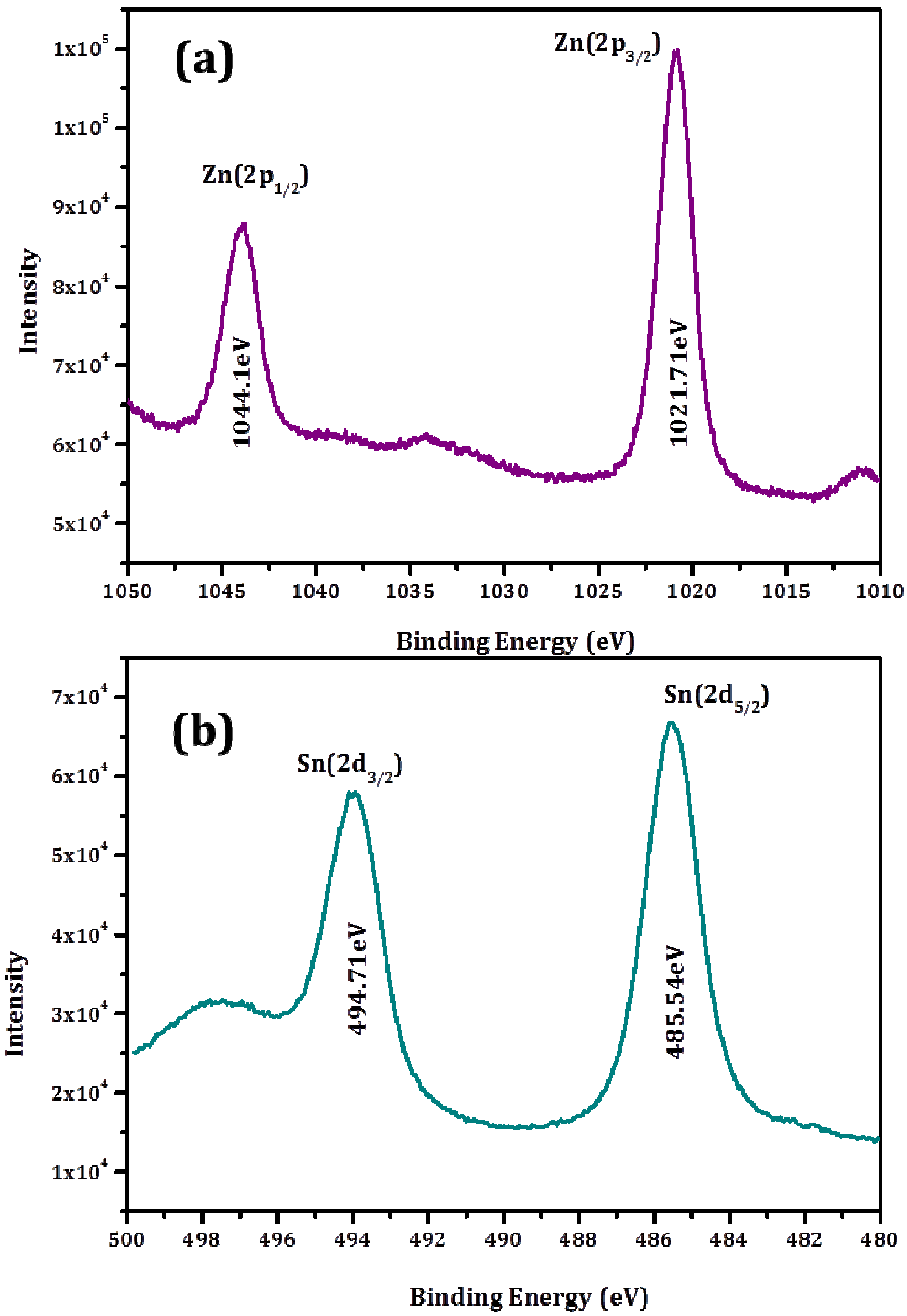

Figure S4FESEM top view and cross sectional view of perovskite solar cell (spiro-MeOTAD/$CH_3NH_3PbI_3$/Zn_2SnO_4-700/Bl-ZSO/FTO) sample deposited at different spin coating speed.Figure (a-h) show photographs of respective sample. (a)as-spun on FTO substrate(b) 2000 (c) 2500 (d) 3000 (e) 3500 (f) 4000 (g) 4500 (h) 5000.

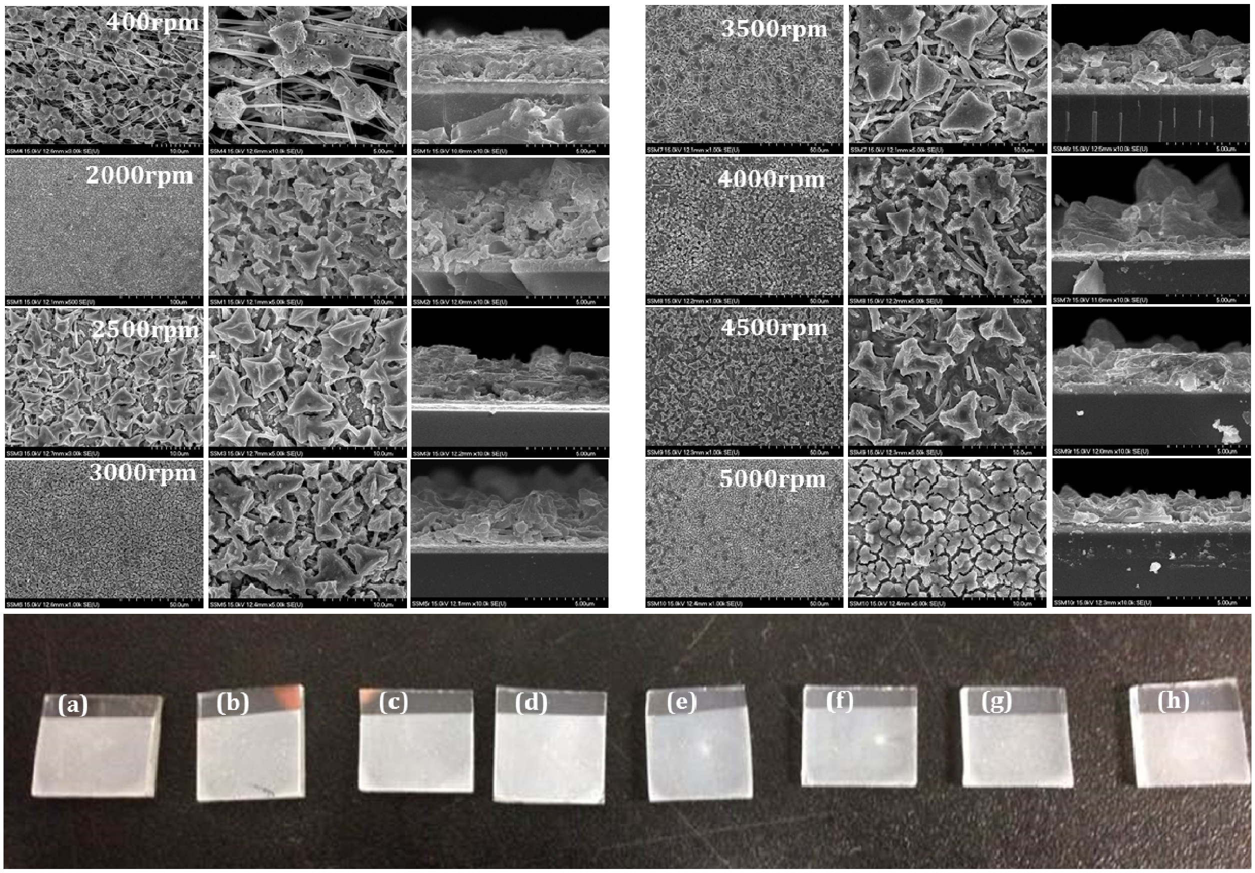

Figure S5XRD pattern of optimized $CH_3NH_3PbI_3$ sample deposited on glass substrate.

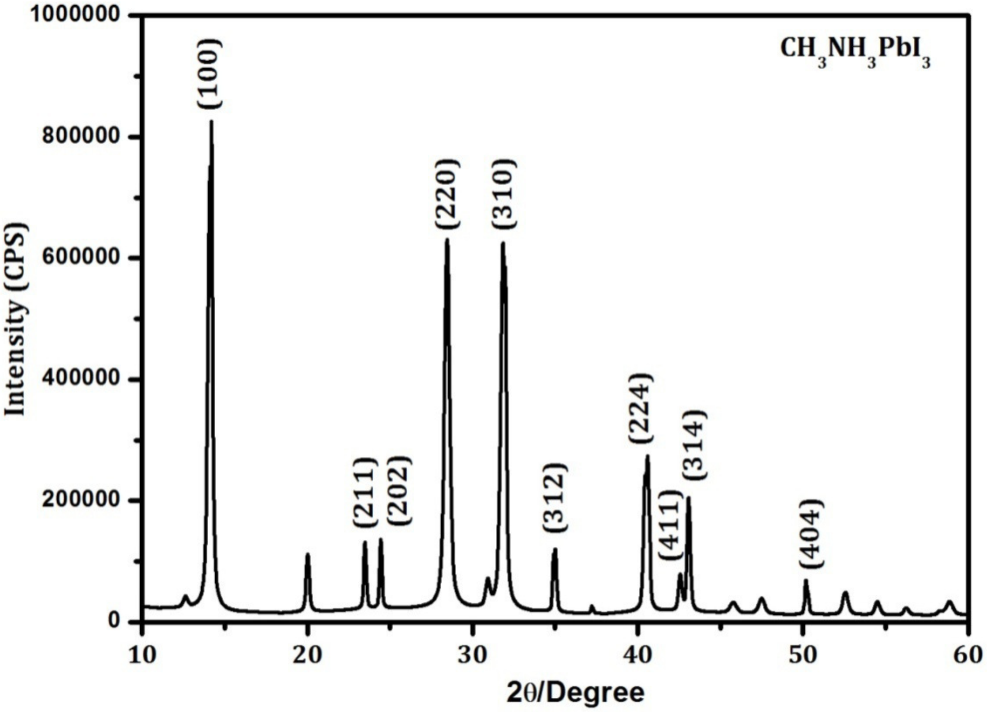

The XRD results reveal $CH_3NH_3PbI_3$ characteristicpeaks at 14.36, 28.61, 32.081,and 40.648 corresponding to the (110),(220), (310) and (224) planes of $CH_3NH_3PbI_3$, respectively. Our calculated lattice parameters for $CH_3NH_3PbI_3$ with a tetragonal unit cell are a=b=8.879Å and c=12.558Å which is in agreement with previous reports a=b=8.883Å and c =12.677Å [1, 2, 3].

References:

[1] kawamura Y., Masghiyama H. & Hasebe K., Structural study on cubic-tetragonal transition of $CH_3NH_3PbI_3$, *J. Phys. Sco. Japn*, **71,** 1694-1697 (2002)

[2] Poglitsch A. & Weber D., Dynamic disorder oin mythylammoniumtrihalogenoplumbates(II) observed by millimeter-wave spectroscopy, J. Chem. Phys., 87, 6373-6377 (1987).

[3] Supasai T., Rujisamphan N., Ullrich K., Chemseddine A.&DittrichTh., Formation of a passivating $CH_3NH_3PbI_3/PbI_2$ interface during moderate heating of$CH_3NH_3PbI_3$ layers, *Appl. Phys. Lett.*103, 183906-3 (2013).

Figure S6 Zn_2SnO_4 nanoparticles synthesized by hydrothermal process. (a-b) FESEM images of Zn_2SnO_4 nanoparticles at different magnification (c) XRD pattern (d) J-V plot of fabricated perovskite device based on Zn_2SnO_4 nanoparticles. Device configuration

FTO/Bl-ZSO/Zn_2SnO_4+$CH_3NH_3PbI_3$/spiro-MeOTAD/Au

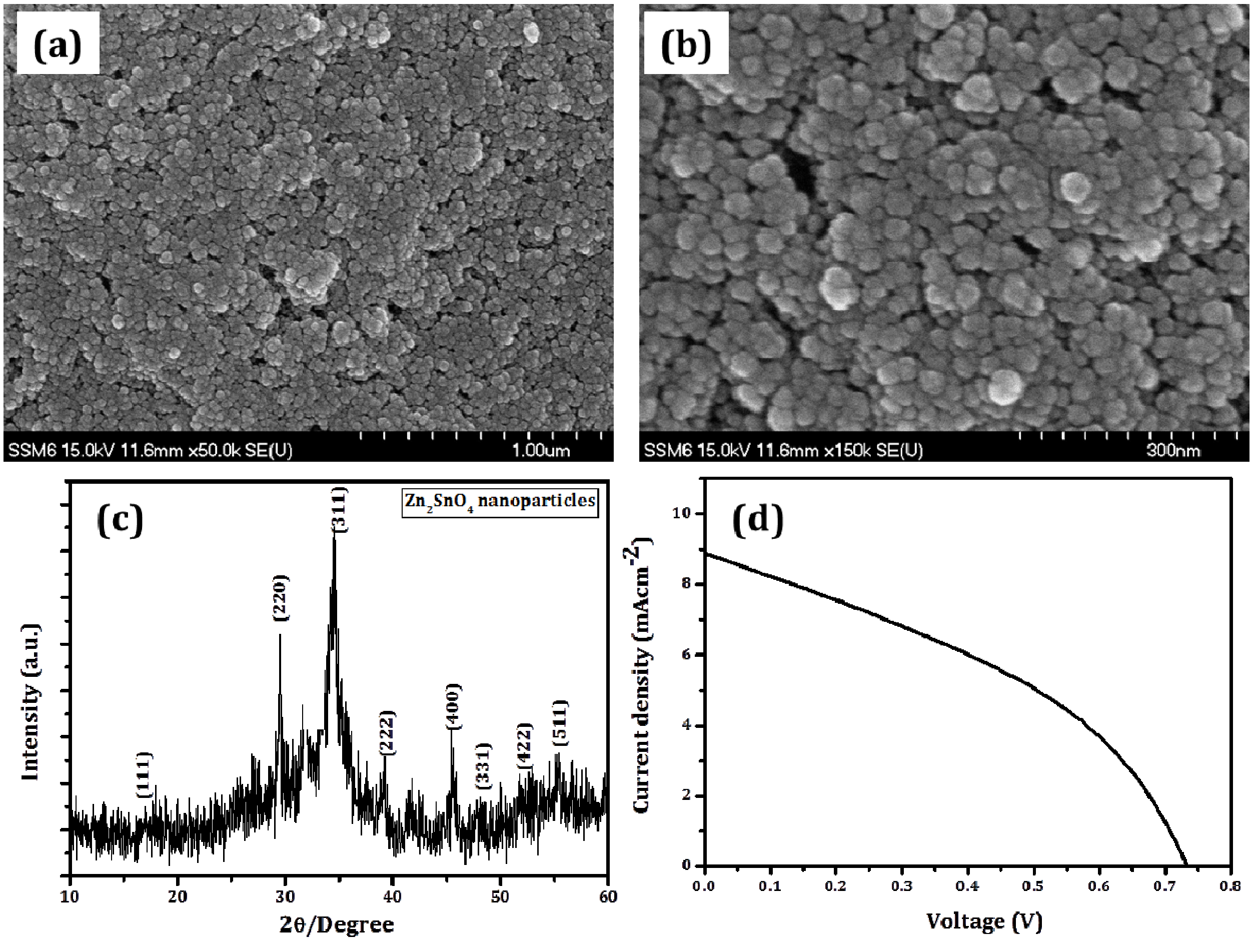

Table S1 Standard and observed d values and their relative intensities of Zn_2SnO_4 nanofibers annealed at 500 and 700°C.

Sr. No.	2θ (Standard)	2θ (Observed)	(hkl) Plane	Standard d (Å) a=8.6574	Observed d (Å) a=8.6586 500°C	Observed d (Å) a=8.6586 700°C	Standard relative intensity I/I_0 (%)	Observed relative Intensity I/I_0 (%) 500°C	Observed relative Intensity I/I_0 (%) 700°C
1	17.772	17.559	111	5.001	5.0241	5.0465	25	17.2	21
2	29.140	29.064	220	3.062	3.0662	3.0699	19	19	20.6
3	34.290	34.279	311	2.613	2.6180	2.6138	100	100	100
4	35.906	35.906	222	2.499	2.4967	2.45048	20	29.9	25.7
5	41.683	41.695	400	2.165	2.1642	2.1644	25	31.7	23.4
6	45.642	45.638	331	1.986	1.9828	-	3	6.7	-
7	51.657	51.446	422	1.768	1.7747	1.7747	7	14.4	10.4
8	55.114	55.076	511	1.665	1.6627	1.6661	30	35.1	33.2
9	60.440	60.454	440	1.5304	1.5308	1.5301	35	53.4	35
10	63.510	-	531	1.4636	1.4638	-	4	6.2	-
11	68.491	-	620	1.3688	-	-	2	-	-
12	71.382	71.296	533	1.3203	1.3203	1.3217	9	13.1	10.1
13	72.337	72.478	622	1.3052	1.3042	1.3030	8	14.8	11.1
14	76.089	76.120	444	1.2499	1.2472	1.2495	3	4.9	4.2
15	78.913	-	711	1.2121	1.2120	-	2	4.5	-
16	83.490	-	642	1.1569	-	-	2	-	-
17	86.232	-	731	1.1270	-	-	12	-	-
18	90.780	-	800	1.1569		-	4	-	-

Note: Dash (-) places observed intensity was extremely low.

Table S2 Optimization Zn_2SnO_4-700photoelectrodes thickness by of spin coating speed.

Sample	Spin coating speed	Thickness (nm) *	V_{OC} (V)	J_{SC} ($mAcm^{-2}$)	FF (%)	η (%)
	2000	1800	0.732	3.56	0.27	0.70
	2500	1200	0.795	5.37	0.31	1.32
	3000	1200	0.853	6.32	0.31	1.65
Zn_2SnO_4-700	3500	1100	0.883	9.56	0.36	3.04
	4000	900	0.896	10.23	0.43	3.94
	4500	600	0.945	10.53	0.48	4.78
	5000	400	0.986	12.68	0.59	7.39

**Thickness measurement of these photoelectrodes is not accurate, since nanofibers are randomly dispersed onto FTO substrate and this is composite of Zn_2SnO_4+$CH_3NH3PbI_3$+HTM layer. However, the mentioned values are average values of 10 devices for same condition.*
